# Suitability of Embedded Liquid Cooling and Heat Generation for Chips

**DOI:** 10.3390/mi15010009

**Published:** 2023-12-20

**Authors:** Jian Zhang, Jiechang Wu, Zhihui Xie, Zhuoqun Lu, Xiaonan Guan, Yanlin Ge

**Affiliations:** 1 School of Power Engineering, Naval University of Engineering, Wuhan 430033, China; 13251586887@163.com (J.Z.); wujiechang1234@163.com (J.W.); luzhuoqun@huhst.edu.cn (Z.L.); guan.xiao@hotmail.com (X.G.); 2 School of Energy and Mechanical and Electrical Engineering, Hunan University of Humanities and Science, Loudi 417000, China; 3 Institute of Thermal Science and Power Engineering, Wuhan University of Technology, Wuhan 430205, China; geyali9@hotmail.com; 4 School of Mechanical and Electrical Engineering, Wuhan University of Technology, Wuhan 430205, China

**Keywords:** chip cooling, embedded cooling, heat distribution, suitability, thermal design

## Abstract

Embedded liquid cooling is a preferred solution for dissipating the heat generated by high-power chips. The cooling capacity and pump power consumption of embedded liquid cooling heat sinks differ significantly between different structures. To achieve an accurate match between cooling capacity and heat dissipation requirements, the selection of a liquid-cooled heat sink should be carefully considered in conjunction with the heat dissipation needs of heat sources in real-world thermal management issues. Based on the manufacturing limitations on chip temperature and microchannel pressure, a composite performance index function was developed to assess the cooling capacity and cooling cost of the heat sink. This allowed for the establishment of an evaluation standard to determine the suitability of embedded liquid cooling and heat sink for the heat source. In this study, the suitability of four microchannel heat sinks with the same feature length and fin volume was evaluated under various thermal load conditions. The results show that the best-suited heat sink changes with variations in the thermal load of the chip. In the example, when the heat source was homogeneous at 100 W, the circular section pin fins have an optimal suitability of 0.928 for Re = 500. When the heat source was a heterogeneous heat source with a power of 100 W, the value of *Θ* was found to be 0.389. Additionally, the optimal suitability of drop section pin fins for Re = 971.5 was determined to be 0.862.

## 1. Introduction

Since Moore introduced “Moore’s Law” in 1965 [1], the degree of integration of chips has increased rapidly, and their characteristics of lightweight, miniaturization, and high power have become evident. Consequently, the thermal load of chips has increased, and the thermal problem has become a key factor affecting the stability and reliability of chips [2,3,4]. The liquid cooling heat sink has become a widely favored chip-heat dissipation solution owing to its small size [5], strong heat dissipation capacity, and easy packaging. To further improve the heat dissipation capacity of the liquid cooling heat sink, several scholars have conducted research on the optimal design of the liquid cooling heat sink [6,7,8,9,10,11,12,13,14,15]. Among them, the embedded heat sink cooling technology shortens the heat dissipation path of the chip from “chip-TIM-packing-shell-TIM-heat sink” to “chip-heat sink-working fluid”, that is, it bypasses the chip packaging and directly cools the hotspot of the chip [16], effectively coping with the more severe heat dissipation challenge caused by the surge during chip integration. Therefore, the research on embedded liquid cooling heat sinks has aroused extensive interest from many scholars, both at home and abroad [17,18,19,20,21,22,23,24,25,26].

It is worth noting that in practical engineering applications, the power density of different chips and the size of hotspot heat flux are different; therefore, it is necessary to design and select the heat sink that satisfies the heat dissipation requirements of the chip, effectively reduces the hotspot temperature, prevents overheating and ablation, and avoids wasting pump power. Chen et al. [27], aiming at solving the problem of non-uniform heating of electronic devices, established a three-dimensional non-uniform rectangular heat-generating body model, taking into account fluid flow and heat transfer, and optimized the aspect ratio of the cooling flow channel by considering the composite function of fire accumulation dissipation rate and pump power consumption as the optimization objective. Danish et al. [28] proposed a type of micro-channel heat sink with a channel-pin fin mixed arrangement to better adapt to the heat dissipation requirements of a non-uniform heat source. For a non-uniform heat source, Lee et al. [29] arranged high-density oblique cluster fins in the area with high heat flux, thus enhancing the local cooling capacity of the hotspot area and improving the suitability of the microchannel heat sink to the non-uniform heat source. Rajalingam et al. [30] strengthened the cooling capacity of the microchannel to the non-uniform heat hotspot region by changing the local channel width. However, there is no quantitative index to evaluate the suitability of heat sinks and heat sources (heat dissipation demand) in the existing literature.

Performance evaluation of a heat sink considers the cooling capacity and cooling cost. During the evaluation of convective heat sink cooling capacity and cooling costs [31], the convective heat transfer coefficient reflects the cooling capacity for a common maximum temperature, heat transfer rate, and Nusselt number; with flowing pressure drop, pump power, the friction factor reflecting the cooling cost, or the two single index is adopted to establish the composite performance index function for comprehensive evaluation. As temperature uniformity is also very important for practical thermal engineering evaluation, this study considers the highest temperature, temperature uniformity, and coolant flow pressure drop as the three factors, and builds the quantitative evaluation degree, i.e., the heat suitability composite performance index function, for the heat sink, taking into account the weighting factor as the demand for cooling capacity, thermal load, and cooling cost requirements.

Based on the aforementioned conditions, this study established four embedded liquid-cooled heat-sink-cooling models. To distinguish the chip power density from the discrete degree of power distribution, the finite element method was applied to the numerical calculation and analysis of the uniform and non-uniform heat sources with different heat sink structures and the inlet Reynolds number. The changing rule of the composite performance index and the suitability of the heat sink and the heat source were studied. This study provides a new method for the optimal design of the chip cooling scheme.

## 2. Model Building

### 2.1. Geometric Model

**Figure 1:**
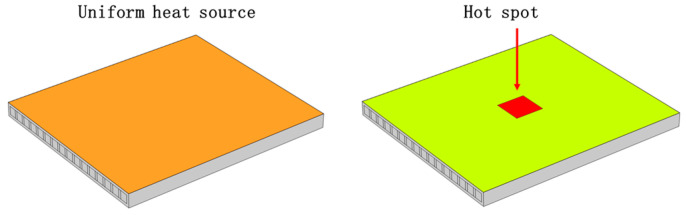
Schematic diagrams of chip power distribution.

Figure 1 shows two power distributions of the chip; the left side of Figure 1 shows uniform distribution of the heat source and the right side of Figure 1 shows the non-uniform distribution of the heat source.

**Figure 2:**
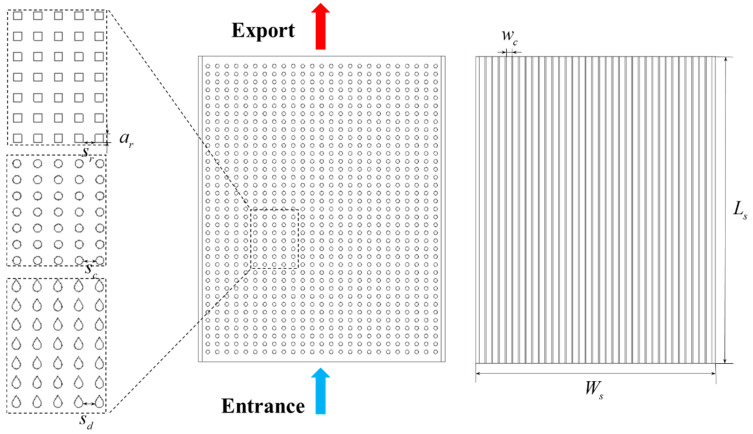
Geometrical structures of the heat sinks.

Figure 2 shows the geometric structures of a rectangular channel heat sink (hereinafter referred to as channel heat sink) with square, round, and drop section pin fin arrays. Referring to the common geometric dimensions of the embedded liquid cooling heat sink [18,24], and considering comparability, the characteristic length of the liquid flow channel in the four types of heat sinks is taken as 0.3 mm and the volume of solid materials in the four types of embedded heat sinks is equal, at 9.9×10^−8^ m^3^. The length, width, and height of the heat sink are 15,000, 13,000, and 1000 μm, respectively, and the thickness of the heat sink wall is 200 µm. The numbers of straight channels and pin fins were 26 and 25, respectively. Table 1 shows the dimensions and parameters of the geometric model [18,24]. Here, the equation for the pin fin contour curve of the drop section is given by,

(1) $$x=0.0675\times \frac{\left(1+\cos(\theta ))\times (2+\cos(\theta )\right)}{5+4\times \cos(\theta )},$$

(2) $$y=\frac{2.4\times (1+\cos(\theta ))\times \sin(\theta )}{(5+4\times \cos(\theta ))\times \sqrt[{2}]{26\times \sqrt[{2}]{13}-70}},$$

where the value range for θ is $\theta \in \left[0,2\pi \right]$.

### 2.2. Physical Model

The liquid coolant used is deionized water. The thermophysical properties of the materials used in this study are listed in Table 2.

In this study, the convective heat transfer problems of four types of embedded liquid cooling heat sinks under two heat sources are studied. The hypotheses are as follows:

(1) Fluid flow and heat transfer are in a steady state, the coolant is incompressible, and the flow state is laminar.
(2) The fluid and solid materials are constant, and the solid heat conduction materials are isotropic.
(3) A no-slip boundary condition is adopted on the flow channel wall.
(4) The dissipation heat caused by radiation heat transfer and viscous dissipation is not considered.

Based on the aforementioned assumptions, the continuity equation for the fluid flow is

(3) ∇⋅u=0,

The momentum conservation equation for the fluid flow is

(4) $$\rho_{f}\left(u\cdot \nabla u\right)=\nabla \cdot [-pI+\mu (\nabla u+(\nabla u{)}^{T})]+F,$$

The energy conservation equation for the fluid flow is

(5) $$\rho_{f}c_{p,f}u\cdot \nabla T_{f}=k_{f}\nabla^{2}T_{f},$$

The energy conservation equation for the solid is

(6) $$\nabla^{2}T_{s}=0,$$

The continuity equation for heat flow and temperature at the interface of the solid and the fluid is

(7) $$k_{s}\frac{\partial T_{s}}{\partial n}=k_{f}\frac{\partial T_{f}}{\partial n},$$

(8) $$T_{s}=T_{f},$$

where $\rho_{f}$ (kg·m^−3^) is the fluid density, u (m·s^−1^) is the fluid velocity vector, *p* (Pa) is the pressure, ***I*** is the identity matrix, F (N) is the volume force vector, $k_{f}$ (W·m^−1^·K^−1^) is the thermal conductivity of the fluid, and $k_{s}$ (W·m^−1^·K^−1^) is the thermal conductivity of the solid.

The boundary conditions are as follows:

(1) Fully developed flow and heat transfer and constant inlet water temperature.
(2) Inlet Reynolds number ranges from 250 to 1500.
(3) The outlet relative pressure is 0 Pa.
(4) Given the heat flow density of each partition of the chip—except for the position in contact with the chip—other outer wall surfaces of the heat sink are adiabatic.

In combination with the above boundary conditions, Equations (3)–(8) are solved to obtain information on the temperature and fluid pressure distributions.

### 2.3. Performance Specifications

$x_{T}$, $x_{f}$, and $x_{P}$ are the normalized chip maximum temperature and temperature uniformity factor between inlet and outlet pressure drops, respectively. $\lambda_{1}$, $\lambda_{2}$, and $\lambda_{3}$ are the weighted factors. The weight coefficient represents the degree of attention paid to the different properties of liquid cooling and heat sink. $\lambda_{1}$ reflects the heat dissipation capacity, $\lambda_{2}$ reflects the uniformity of heat dissipation, and $\lambda_{3}$ reflects the cost of heat dissipation.

#### 2.3.1. Maximum Temperature

In the actual operation of the chip, the highest temperature usually represents the chip’s threshold temperature. When the temperature of the chip exceeds the threshold temperature, the chip actively reduces its operating frequency to minimize heat generation. Lowering the maximum temperature of the chip helps it to continue functioning at higher frequencies and enhances its overall performance. The lower the maximum temperature, the greater the cooling capacity of the heat sink.

#### 2.3.2. Temperature Uniformity Factor

The uniform temperature distribution is beneficial for reducing signal transmission delay in the chip and preventing local warping caused by thermal stress due to excessive temperature gradients. The temperature uniformity factor can describe the evenness of temperature distribution on the chip. Its definition can be expressed as [32].

(9) $$f_{T}=\sqrt{\int \int \int (T-{\bar{T}}_{s}{)}^{2}dxdydz/V_{s}},$$

The average temperature ${\bar{T}}_{s}/K$ is defined as

(10) $${\bar{T}}_{s}=\int \int \int Tdxdydz/V_{s},$$

where $V_{s}/m^{3}$ is the total volume of the computing domain. As the uniformity of temperature distribution on the interface between the heat source and heat sink in this model has more practical significance, the actual calculation domain of temperature uniformity and average temperature in this study is the upper surface of the heat sink. The smaller the temperature uniformity factor value, the more uniform the temperature distribution.

#### 2.3.3. Inlet and Outlet Pressure Drop

The inlet and outlet pressure drop of coolant Δp is the pressure drop caused by energy loss in the flow process, which is defined as

(11) $$\Delta p=p_{\mathrm{in}}-p_{\mathrm{out}},$$

where $p_{\mathrm{in}}$ and $p_{\mathrm{out}}$ are the average pressures of the inlet and outlet sections of the coolant, respectively. The smaller the coolant flow Δp, the smaller the energy loss, that is, at the same mass flow rate, less pump power is consumed.

#### 2.3.4. Composite Performance Indicators

The maximum temperature and flow pressure drop reflect the cooling capacity and cooling cost of the heat sink, respectively. Temperature uniformity is also a significant concern in the cooling of electronic and optoelectronic devices, as well as in other practical applications. To accurately assess the convective cooling performance and cooling cost in practical engineering problems, a weighted composite performance index function was developed. This function takes into account the maximum temperature, temperature uniformity, and flow pressure drop of the coolant. Since the dimensions of the three elements are different and the Min–Max standardization method accurately represents the proportional relationship between the corresponding data and the maximum and minimum values in the dataset, it is beneficial for describing the suitability between the heat sink and heat source after weighting. Therefore, the Min–Max standardization method is chosen to normalize the three elements.

Microchannel heat sinks are designed with pressure limitations in mind. Because the microchannel heat sink channels are small, the liquid flow resistance is high. If the fluid pressure is too high, the microchannel heat sink may be damaged or develop leaks. Therefore, a reasonable flow and pressure range is usually established in the design to ensure the normal operation and efficient cooling of the microchannel heat sink. At the same time, users also need to pay attention to controlling the inlet and outlet pressures when using the heat sink to avoid high pressure. This is important to ensure the longevity of the heat sink. Please refer to the document titled “Multichannel Positive and Negative Pressure Controller Solution for Microfluidic Chip” provided by Shanghai Yiyang Industrial. Positive and negative pressure control range: absolute pressure 1 Pa–0.5 MPa. When the maximum pressure drop of a heat sink exceeds 0.5 MPa, the heat sink is considered unqualified. The ${\Delta P}_{max}$ is 0.5 MPa. Natural convection is a low-energy cooling method that does not require external pressure. As a result, it can be an ideal cooling solution in certain situations. The ${\Delta P}_{min}$ is 0 Pa.

The exact value of the optimum operating temperature for a chip is usually determined by the chip manufacturer and given in the chip specifications. Different chip models may have different optimum operating temperature ranges, which are listed in the specifications. The optimal operating temperature range for a chip is usually determined by the chip design, materials, and process. In practice, to ensure the performance and life of the chip, the operating temperature of the chip should be kept within the optimum operating temperature range given in the specifications. The maximum heat dissipation capacity should keep the operating temperature of the chip at the lower limit of the optimal operating temperature of the chip. When the cooling performance of the heat sink makes the chip operating temperature lower than the lower limit of the optimal operating temperature, it is always marked as 1. In this study, $T_{min}$ is defined at 338.15 K.

When the operating temperature of the chip exceeds its critical damage temperature, it can result in chip damage or performance degradation. Therefore, the heat sink must ensure that the temperature of the chip during operation is always below its critical damage temperature. If the cooling capacity of the heat sink is not sufficient to keep the chip temperature below the critical damage temperature, the heat sink is disqualified. In this paper, $T_{max}$ is 373.15 K.

The maximum temperature uniformity factor is defined as the maximum temperature uniformity factor obtained under the same thermal load. The minimum temperature uniformity factor is defined as the minimum temperature uniformity factor obtained under the same thermal load.

(12) $$\Phi =1-\lambda_{1}x_{T}-\lambda_{2}x_{f}-\lambda_{3}x_{P},$$

(13) $$x_{f}=\frac{T_{i}-T_{min}}{T_{max}-T_{min}},$$

(14) $$x_{f}=\frac{f_{T}-{\left(f_{T}\right)}_{min}}{[{\left(f_{T}\right){-\left(f_{T}\right)}_{min}]}_{max}},$$

(15) $$x_{P}=\frac{\Delta P-{\Delta P}_{min}}{{\Delta P}_{max}-{\Delta P}_{min}},$$

where $x_{T}$, $x_{f}$, and $x_{P}$ are the normalized chip maximum temperature, temperature uniformity factor, and inlet and outlet pressure drops, respectively. $\lambda_{1}$, $\lambda_{2}$, and $\lambda_{3}$ are the weighted factors.

#### 2.3.5. Power Dispersion

In practice, the power distribution of the chip changes according to the different tasks of the processor. To describe the power distribution of the heat source, this study proposes power dispersion to reflect the characteristics of chip power distribution Θ, and its construction is as follows:

(16) $$\Theta =\frac{\sum \left|(P_{i}-\bar{P}\right)S_{i}|}{S_{0}}$$

where $P_{i}$ is the average power density of region *i*, $\bar{P}$ is the average power density of the chip, $S_{i}$ is the area of region *i*, and $S_{0}$ is the total area of the chip. When Θ is equal to zero, the reservoir is uniform.

## 3. Results and Discussion

**Figure 3:**
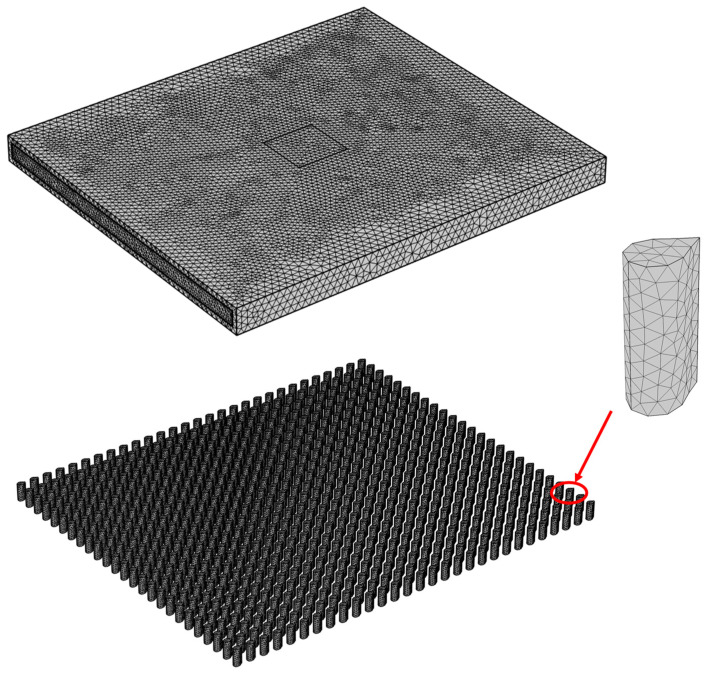
Schematic diagram of the mesh division.

**Figure 4:**
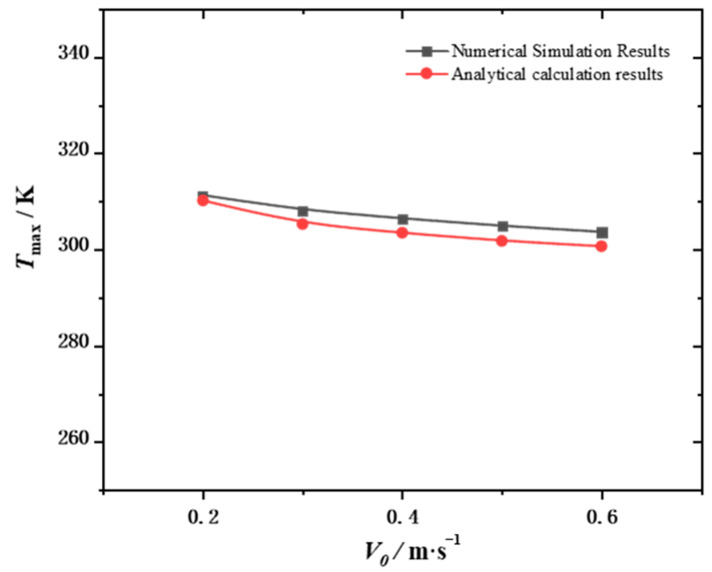
Model validity verification.

In this study, COMSOL Multiphysics5.6 was used to solve Equations (3)–(8) under the corresponding boundary conditions. To ensure the accuracy of the calculation, the independence of the grid was verified. The grid numbers were 377,988, 613,745, 1,186,150, 3,412,455, and 8,621,621, corresponding to 307.70, 308.24, 310.79, 311.57, and 312.19 K, respectively. The relative errors were 1.44%, 1.27%, 0.45%, and 0.20%, respectively. The grid numbers 613,745, 1,186,150, 3,412,455, and 8,621,621 correspond to 142,945, 131,562, 121,960, and 118,460 Pa, respectively, with relative errors of 20.7%, 11.1%, and 3.0%, respectively. Considering the computing efficiency, the partition strategy with mesh number 3,412,455 was selected. Figure 3 shows the grid division diagram of the model. To further verify the accuracy of the numerical model, this study used the analytical method reported in a previous study [30] to derive the analytical formula for the thermal resistance of the heat sink of the pin fins and obtain the analytical solution for the maximum temperature of the heat sink. The comparison with the numerical simulation results in this study is shown in Figure 4.

### 3.1. Variation in Heat Sink Suitability with Thermal Load under Uniform Heat Source

**Figure 5:**
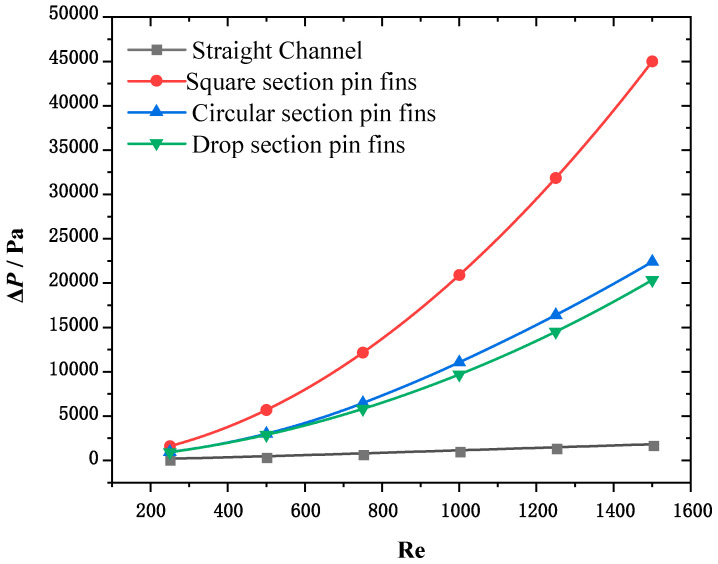
Variation in Δp with the inlet Reynolds number.

**Figure 6:**
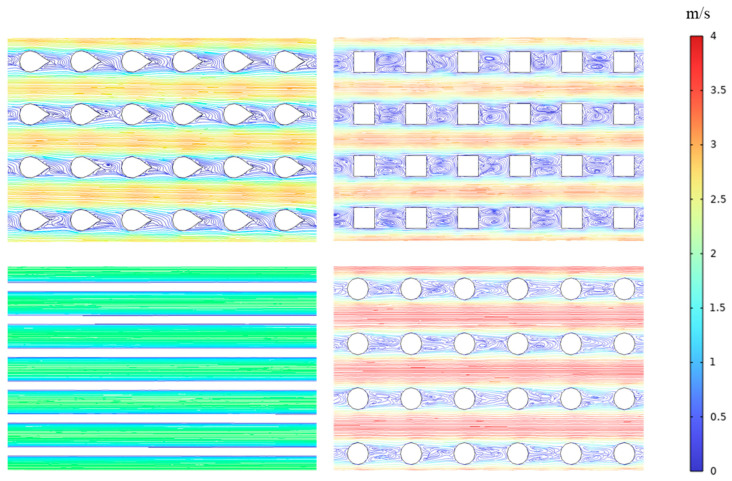
Streamline diagram.

Figure 5 shows the variations Δp in the four heat sinks with the inlet Reynolds number. Figure 6 shows the internal flow diagrams of the four heat sinks when the inlet Reynolds number is 1500.

It can be observed from the figure that the heat sink of the pin fin with square section Δp is the largest and has the strongest hindering effect on coolant flow. This is because the flow face of the square section pin fins is flat, causing significant fluid separation when bypassing them. As the fluid separation continues towards the rear area of the pin fins, it creates a separation zone that leads to significant local resistance. The surface of the circular pin fin is curved, and the degree of fluid bypassing the circular pin fins is smaller than that of the fluid bypassing the square pin fins. The spacing between the circular pin fins is smaller than that of the square pin fins. Therefore, circular section pin fins have less resistance to fluid flow than square section pin fins. The pin fins in the drop section have a streamlined structure and there is no separation in the boundary layer. Therefore, the pin fins in the drop section have less hindrance on the fluid compared to the pin fins in the circular section.

**Figure 7:**
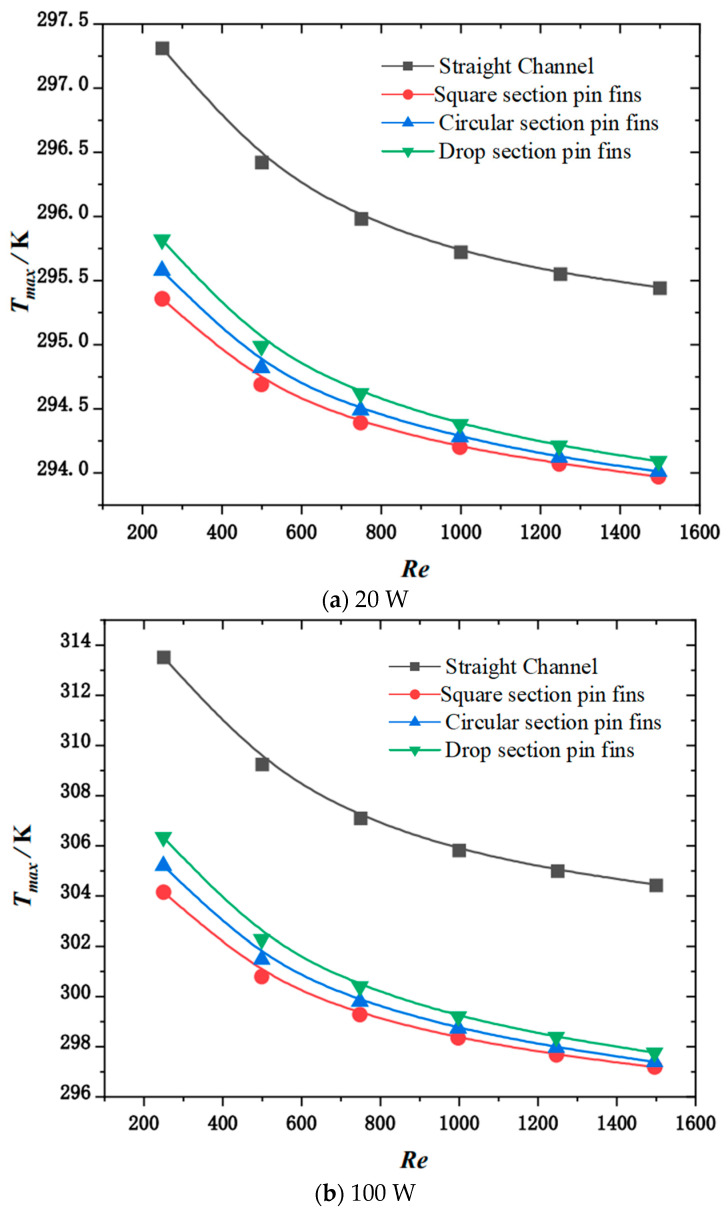

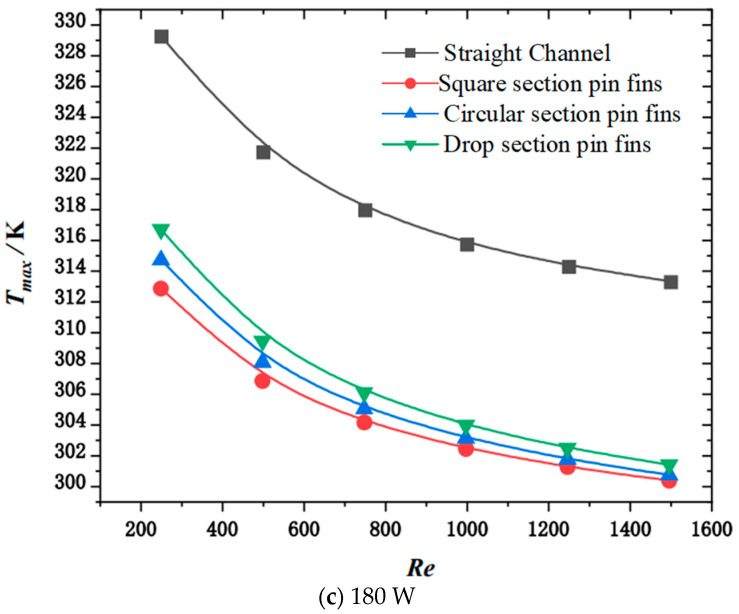
Variation in $T_{max}$ with channel type and inlet Reynolds number under a uniform heat source.

Figure 7 shows the variation rules for $T_{max}$ with changes in the Reynolds number and channel type under the conditions of uniform thermal load at different powers. It can be seen from the figure that, for the four structures, the heat sink values corresponding to $T_{max}$ decrease as the Reynolds numbers increase. Additionally, the change curve gradually levels off. Under the same heat source load, the $T_{max}$ values for the heat sink with Reynolds numbers are close to each other. It can be seen from the figure that the heat sink of the rectangular section of the needle pin fin has the strongest hindering effect on the coolant flow and the strongest heat dissipation capacity. This is due to the fact that the face of the rectangular needle pin fin is flush, the fluid is highly separated when bypassing the rectangular needle pin fin and the fluid disturbance is stronger. At the same time, the contact area of the rectangular needle pin fin is the largest, thus the heat dissipation capacity is strongest.

**Figure 8:**
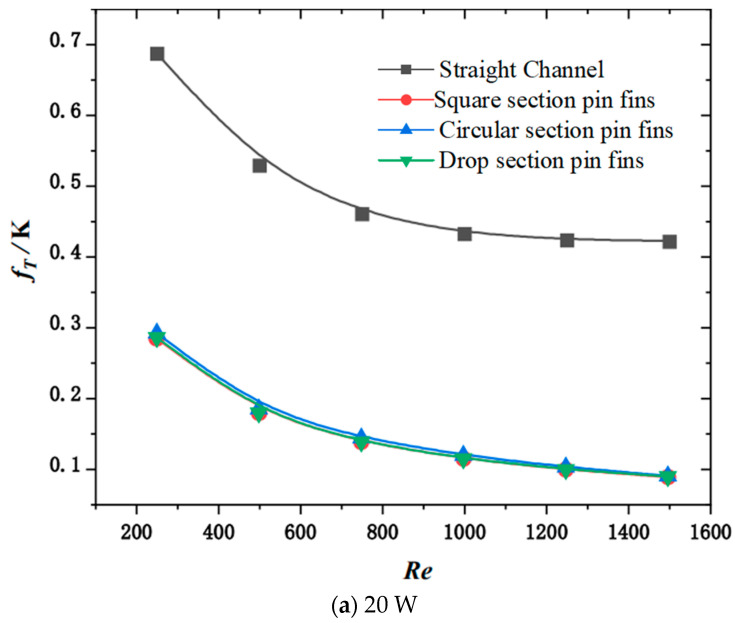

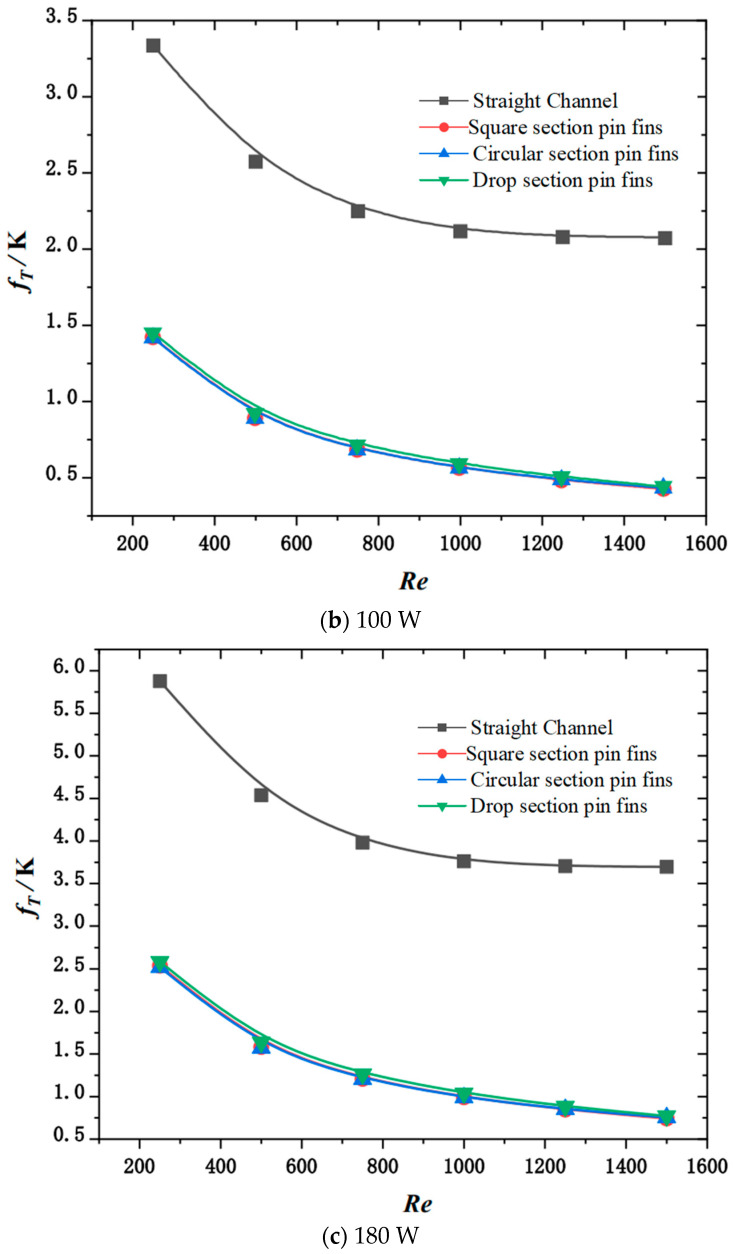
Variation in $f_{T}$ with channel type and inlet Reynolds number under uniform heat source.

Figure 8 shows the variation rules for $f_{T}$ with the change in Reynolds number and channel type under uniform thermal loads at different powers. It can be seen from the figure that, for the four structures, the heat sink values corresponding to $f_{T}$ decrease as the Reynolds number increases. Additionally, the change curve gradually levels off. Under the same heat source load, the $f_{T}$ values of the heat sink with Reynolds numbers are close to each other.

It can be observed from the figure that, with the increase in the thermal load of the chip, the corresponding $T_{max}$ and $f_{T}$ of the four heat sinks increase. When the heat source load is 20 W, $T_{max}$ and $f_{T}$ of the heat sink of the channel are 3.35 K and 0.601 K higher than those of the pin fin with the square section. When the heat source load is 180 W, $T_{max}$ and $f_{T}$ of the heat sink of the channel are 28.97 K and 5.136 K higher than those of the square section pin fin. That is, with increasing heat source load, the difference between the $T_{max}$ and $f_{T}$ of the heat sink of the channel and the heat sink of the pin fin of the square section increase gradually. However, when the thermal loads are 20 and 180 W, the pump power consumed by the channel heat sink and the square section pin fins heat sink remains unchanged. The $T_{max}$ and $f_{T}$ corresponding to the heat sink of the pin fins of the circular section are smaller than those of the pin fins of the drop section. This is because the disturbance effect of the cylindrical structure on the fluid is stronger than that of the streamlined drop structure; therefore, the cooling capacity of the circular section pin fin heat sink is slightly stronger than that of the drop section pin fin heat sink.

**Figure 9:**
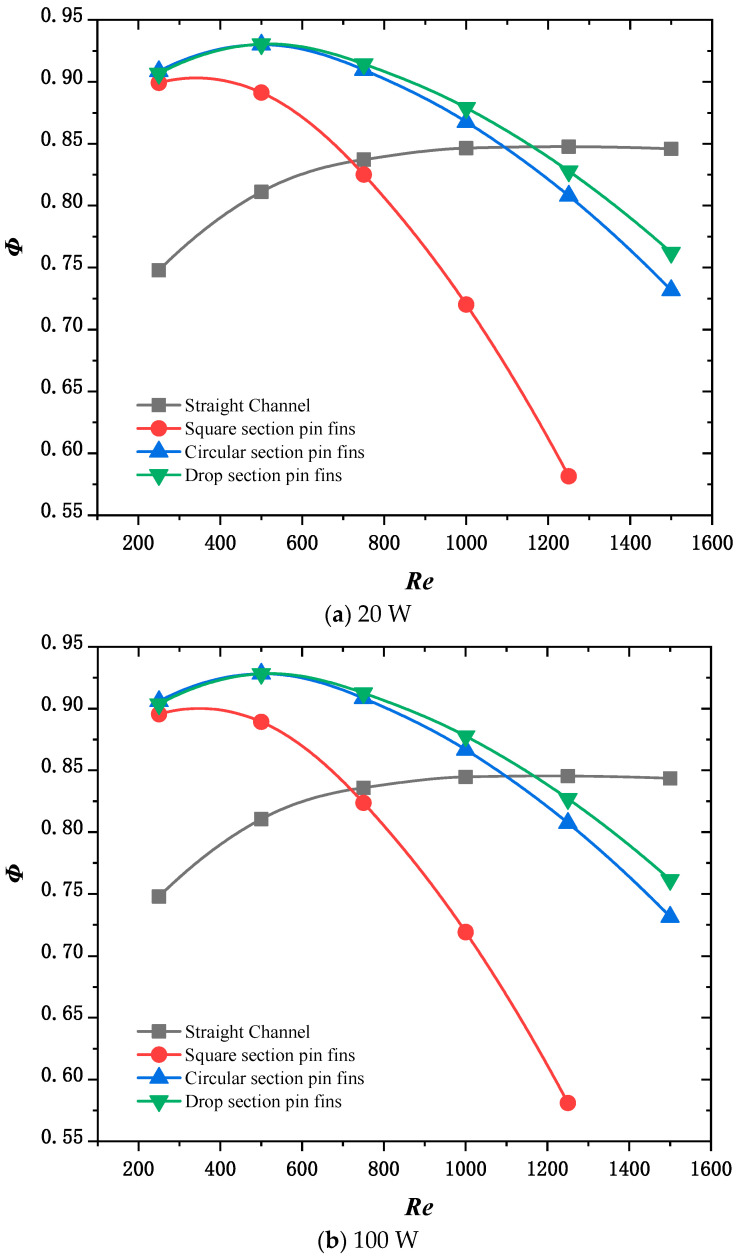

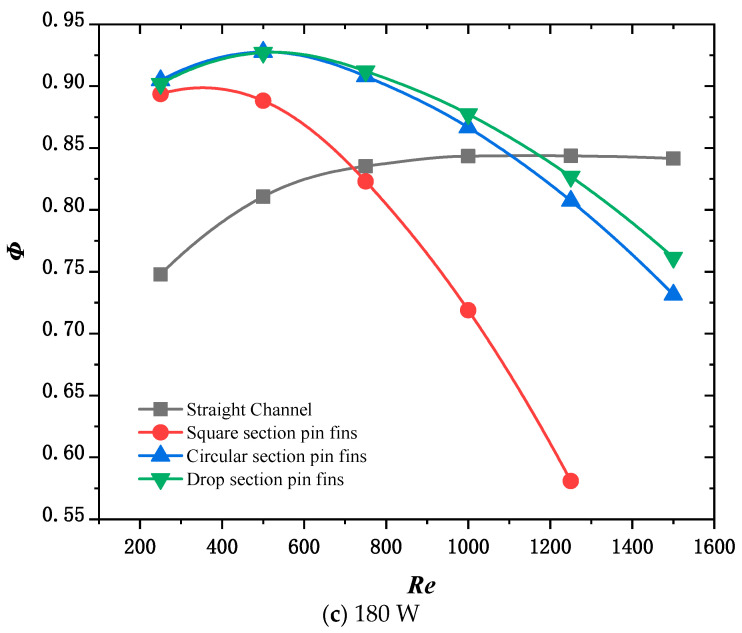
Variation in the composite performance index Φ with inlet Reynolds number.

Figure 9 shows the variation in the composite performance index with inlet Reynolds numbers under thermal loads of 20, 100, and 180 W, respectively, when $\lambda_{1}=0.25$, $\lambda_{2}=0.25$, and $\lambda_{3}=0.5$. The reason why these graphs tend to be close is that, under the condition of a uniform heat source, multiple cooling capacities of a heat sink can effectively cool the heat source. In the composite function of the evaluation method, the main dominant term is the pressure drop of the heat sink at the inlet and outlet. In other words, when the heat sink’s capacity to dissipate heat suits the heat source’s heat dissipation, it is important to consider the pressure drop at the inlet and outlet.

Because the heat load of a homogeneous heat source is small, the trend in the change in heat sink adaptation under different power groups is close. After considering heat dissipation capacity, temperature uniformity, and heat dissipation cost, it was found that heat sink adaptation of the drop section is better when the Reynolds number is about 400.

### 3.2. Variation in Heat Sink Suitability with Thermal Load under Non-Uniform Heat Source

**Figure 10:**
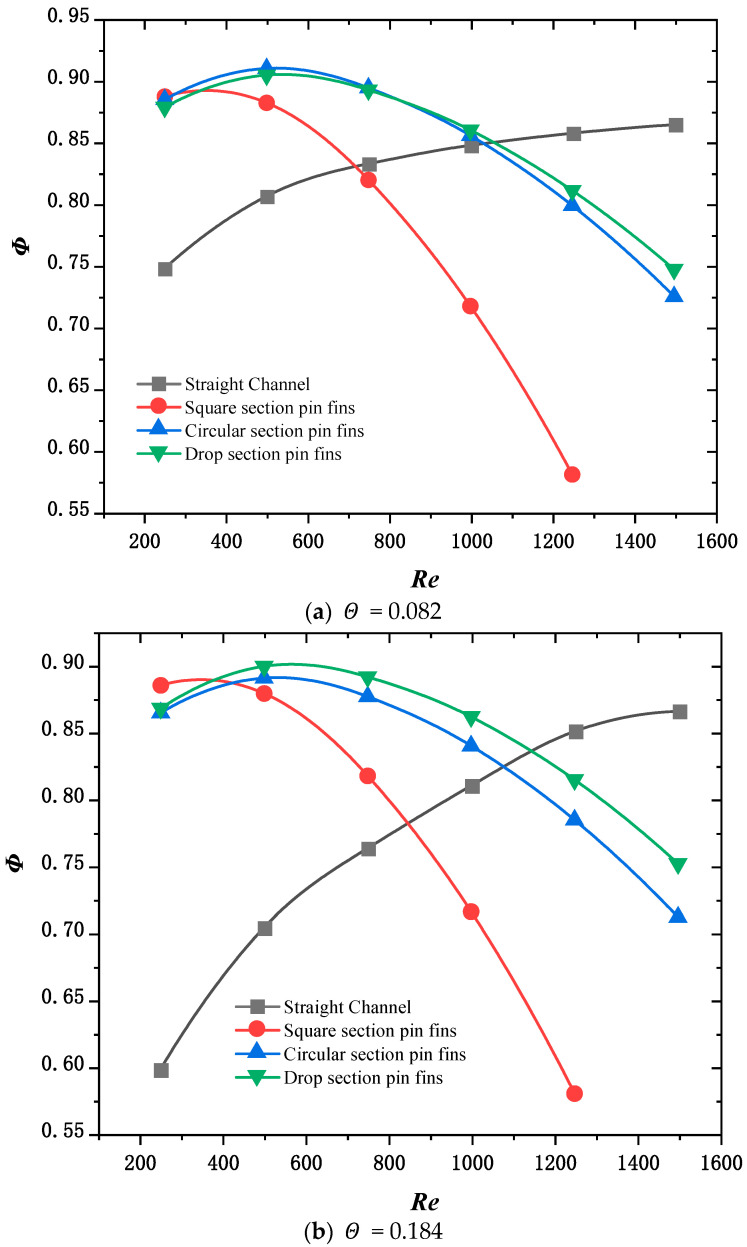

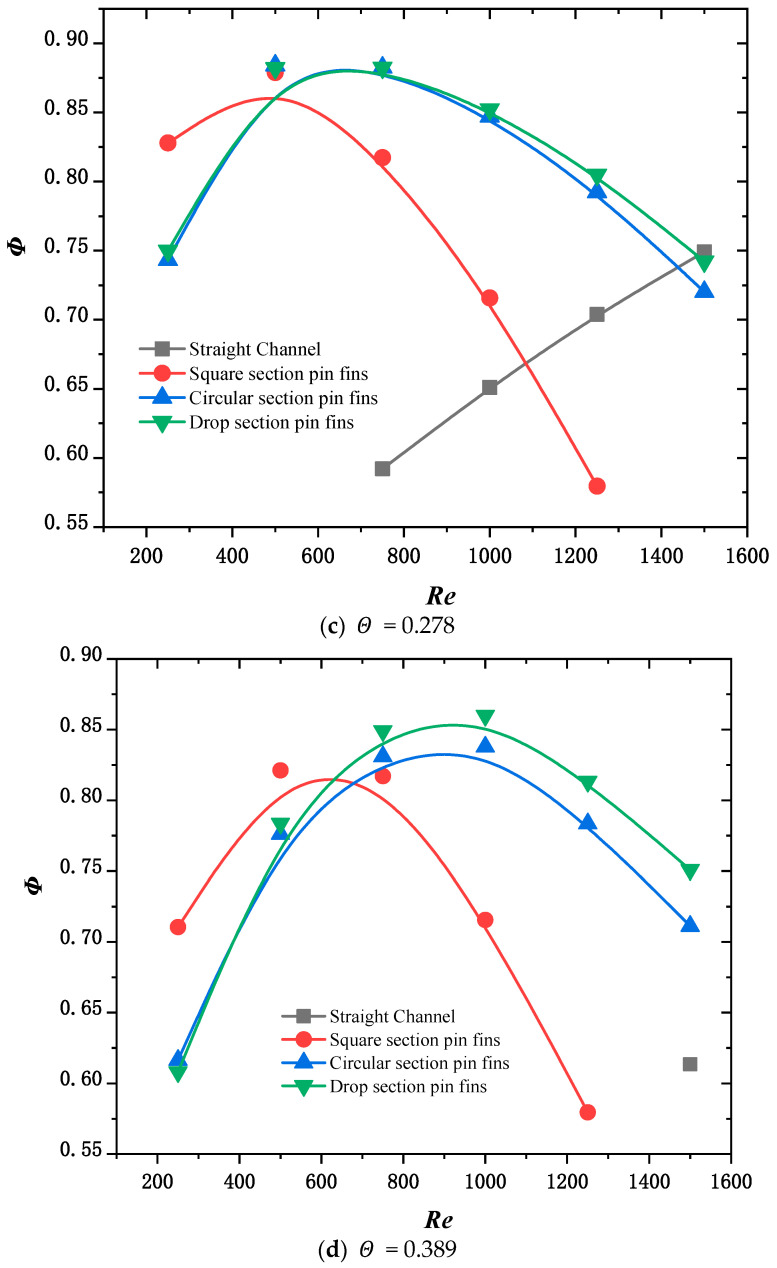
Variation in composite performance index Φ with inlet Reynolds number.

Figure 10 shows the variation Φ in the composite performance index with the inlet Reynolds number for non-uniform heat sources with 100 W power and power dispersion Θ of 0.082, 0.184, 0.278, and 0.389.

The weight of the composite performance index Φ is like that of the uniform heat source. The figure shows that when the power dispersion Θ is 0.082, 0.184, or 0.278, Φ of the heat sink of the pin fin of the circular and drop-shaped sections first increase and then decrease with the increase in the inlet Reynolds number. When the power dispersion Θ is 0.389, Φ of the heat sink of the channel, circular section, and drop section increases with increasing Reynolds numbers between 250 and 1500. At the same time, the composite performance indexes Φ of the channel heat sink range from 250 to 1500, which are lower than those of the pin fin heat sink. This is because as the thermal load of the heat source increases when the power dispersion Θ is 0.389, the pressure drop loss caused by the increase in the Reynolds numbers of the three structures is less than the benefit generated by the increase in cooling capacity. Moreover, the cooling capacity of the channel heat sink cannot fulfill the corresponding cooling demand; therefore, Φ of the channel heat sink is significantly smaller than the micro pin fin heat sink. With increasing Reynolds numbers, the heat sink of pin fins of the square section Φ first increases and then decreases. This is because the pressure drop loss caused by the increase in the Reynolds number caused by the heat sink of the pin fin with the square section is greater than the gain caused by the increase in the cooling capacity.

## 4. Conclusions

In this study, four embedded cooling heat sink models with equal characteristic lengths and total volume of heat sink fins are established to solve the suitability problem between the design of the embedded liquid cooling heat sink structure and the heat source. The composite performance index function is constructed taking into account the cooling capacity (temperature and temperature uniformity) and the cooling cost (pressure drop) of the channel. The model is simulated using the finite element method for uniform and non-uniform heat sources. The following conclusions can be drawn based on the results.

(1) For equal characteristic lengths and volumes, the heat sink of the rectangular channel has the weakest cooling capacity and the least pressure drop; in the structure of the heat sink of the pin fin, the square section fin has the strongest cooling capacity and the largest pressure drop, while the drop section fin has the weakest cooling capacity and the least pressure drop.
(2) The composite performance index function value of the heat sink first increases and then decreases with the Reynolds number, and there exists an optimal inlet Reynolds number to maximize the composite performance index. Further, as the thermal load of the heat source increases, the demand of the heat source for the cooling capacity of the channel increases and the optimal inlet Reynolds number corresponding to the maximum composite performance index also increases.
(3) The suitability laws for different internal fin shapes and inlet Reynolds numbers under different heat source loads were analyzed, the optimal design was obtained by comparing multiple schemes, and a new optimization design method for chip heat dissipation was established.

## Figures and Tables

**Table 1:** Size parameters of the geometry model [18,28].

Geometry	Expression
$\mathrm{Heat}\;\mathrm{sink}\;\mathrm{length}\;L_{s}$	15,000 µm
$\mathrm{Heat}\;\mathrm{sink}\;\mathrm{width}\;W_{s}$	13,000 µm
$\mathrm{Heat}\;\mathrm{sink}\;\mathrm{height}\;H_{s}$	1000 µm
$\mathrm{Heat}\;\mathrm{sink}\;\mathrm{wall}\;\mathrm{thickness}\;w_{wall.s}$	200 µm
$\mathrm{Side}\;\mathrm{length}\;\mathrm{of}\;\mathrm{square}\;\mathrm{section}\;\mathrm{pin}\;\mathrm{fins}\;a_{r}$	200 µm
$\mathrm{Number}\;\mathrm{of}\;\mathrm{rows}\;\mathrm{of}\;\mathrm{square}\;\mathrm{section}\;\mathrm{pin}\;\mathrm{fins}\;N_{r1}$	25
$\mathrm{Number}\;\mathrm{of}\;\mathrm{columns}\;\mathrm{of}\;\mathrm{square}\;\mathrm{section}\;\mathrm{pin}\;\mathrm{fins}\;N_{r2}$	29
$\mathrm{Channel}\;\mathrm{width}\;w_{c}$	300 µm
$\mathrm{Channel}\;\mathrm{wall}\;\mathrm{thickness}\;w_{wall,c}$	$\frac{N_{r1}\times N_{r2}{a_{r}}^{2}}{L_{s}\times (N_{c}-1)}$
$\mathrm{Number}\;\mathrm{of}\;\mathrm{channels}\;N_{c}$	26
$\mathrm{Diameter}\;\mathrm{of}\;\mathrm{pin}\;\mathrm{fins}\;\mathrm{with}\;\mathrm{circular}\;\mathrm{section}\;r_{0}$	$\sqrt{\frac{N_{r1}\times N_{r2}{a_{r}}^{2}}{N_{y1}\times N_{y2}\pi }}$
$\mathrm{Number}\;\mathrm{of}\;\mathrm{rows}\;\mathrm{of}\;\mathrm{circular}\;\mathrm{section}\;\mathrm{pin}\;\mathrm{fins}\;N_{y1}$	25
$\mathrm{Number}\;\mathrm{of}\;\mathrm{columns}\;\mathrm{of}\;\mathrm{circular}\;\mathrm{section}\;\mathrm{pin}\;\mathrm{fins}\;N_{y2}$	37
$\mathrm{Number}\;\mathrm{of}\;\mathrm{rows}\;\mathrm{of}\;\mathrm{drop}\;\mathrm{section}\;\mathrm{pin}\;\mathrm{fins}\;N_{d1}$	25
$\mathrm{Number}\;\mathrm{of}\;\mathrm{columns}\;\mathrm{of}\;\mathrm{drop}\;\mathrm{section}\;\mathrm{pin}\;\mathrm{fins}\;N_{d2}$	31

**Table 2:** Thermophysical parameters [17].

Material	*ρ* [Kg·m^−3^]	*c_p_* [J·Kg^−1^·K^−1^]	*k* [W·m^−1^·K^−1^]	*μ* [Pa·s]
Silicon	2330	712	148	-
Deionized water	998.2	4182	0.6	0.001003

## Data Availability

The data presented in this study are available on request from the corresponding author.
